# A Rare Case of a Sickle Cell Patient With Post Endoscopic Retrograde Cholangiopancreatography (ERCP) Pancreatitis and Pseudoaneurysm Formation: An Association Worth Exploring

**DOI:** 10.7759/cureus.21780

**Published:** 2022-01-31

**Authors:** Vincent Wong, Hasan Ali, Kamal Amer, Sushil Ahlawat

**Affiliations:** 1 Internal Medicine-Pediatrics, Rutgers University New Jersey Medical School, Newark, USA; 2 Internal Medicine, Rutgers University New Jersey Medical School, Newark, USA; 3 Gastroenterology and Hepatology, Rutgers University New Jersey Medical School, Newark, USA

**Keywords:** pancreatic pseudoaneurysm, post-endoscopic retrograde cholangiopancreatography pancreatitis, sickle cell disease (scd), post ercp pancreatitis, visceral artery pseudoaneurysm

## Abstract

Pancreatitis is commonly seen with alcohol use and gallstones, but it can be a post-procedural complication from endoscopic retrograde cholangiopancreatography (ERCP). Inflammation of the pancreas can lead to pseudoaneurysm formation, which is rare but extremely dangerous if ruptures, with high mortality and morbidity. Sickle cell disease can also cause vascular injury from repeated vaso-occlusion, inflammation, and ischemia. Here, we present a case of a 27-year-old patient with sickle cell disease who underwent ERCP for stent placement for gallstones and subsequently developed pancreatitis complicated by pseudoaneurysm formation of the inferior pancreaticoduodenal artery (IPDA) that was managed by endovascular embolization.

## Introduction

Pancreatitis can occur in patients with alcohol use disorder, cholelithiasis, hypertriglyceridemia, and as a post-procedural complication of endoscopic retrograde cholangiopancreatography (ERCP) [1]. It causes episodic abdominal pain often requiring hospitalization [1,2]. As the pancreatic parenchyma and vasculature get repeatedly inflamed, it can lead to necrosis, abscess formation, pseudocysts, and rarely pseudoaneurysms [1,3,4]. Pseudoaneurysms are contained hematomas within the external adventitia of the vessel after the compromise of the muscular layer, which can carry a 90% mortality rate if the vessel wall ruptures [2-4]. Swift recognition and rapid interventions are needed if suspected, for the high risk of exsanguination from brisk, repetitive hemorrhage [3]. Because of the close proximity of the pancreas to blood vessels, pancreatitis can affect the branches of the celiac and pancreaticoduodenal arteries [5]. Sickle cell disease can cause vascular injury as well and has been associated with cerebral aneurysms [6]. Here, we will describe a case of pseudoaneurysm formation in a sickle cell patient after ERCP-induced pancreatitis.

## Case presentation

A 27-year-old male with a history of HbSS disease, multiple vaso-occlusive crises, transient ischemic attacks, bilateral retinal artery occlusions, and splenic sequestration requiring splenectomy at one year old presented with acute persistent epigastric and right upper quadrant (RUQ) abdominal pain for three days. He was experiencing intermittent bouts of abdominal pain, exacerbated by meals, for the past month. His symptoms continued and became associated with nausea and decreased appetite. He denies any fever, chills, chest pain, shortness of breath, vomiting, or diarrhea. This was different from his usual sickle cell crises pain which is a pain in his back and his legs. He reports being compliant with hydroxyurea and deferasirox for iron overload syndrome from monthly red blood cell transfusions.

**Figure 1:**
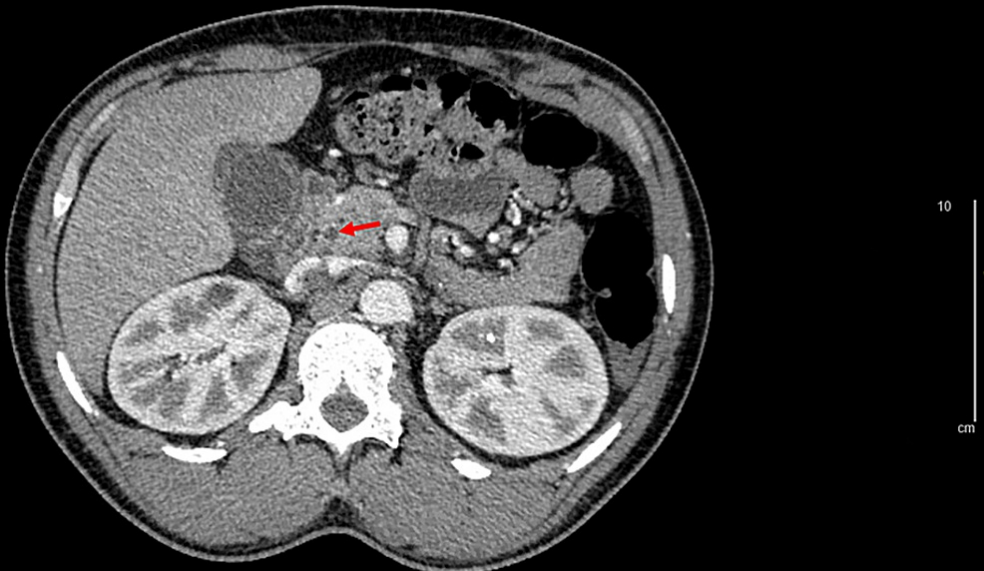
CT scan on admission showing 0.6 cm dilation of the common bile duct (red arrow) at 1.42x magnification.

**Figure 2:**
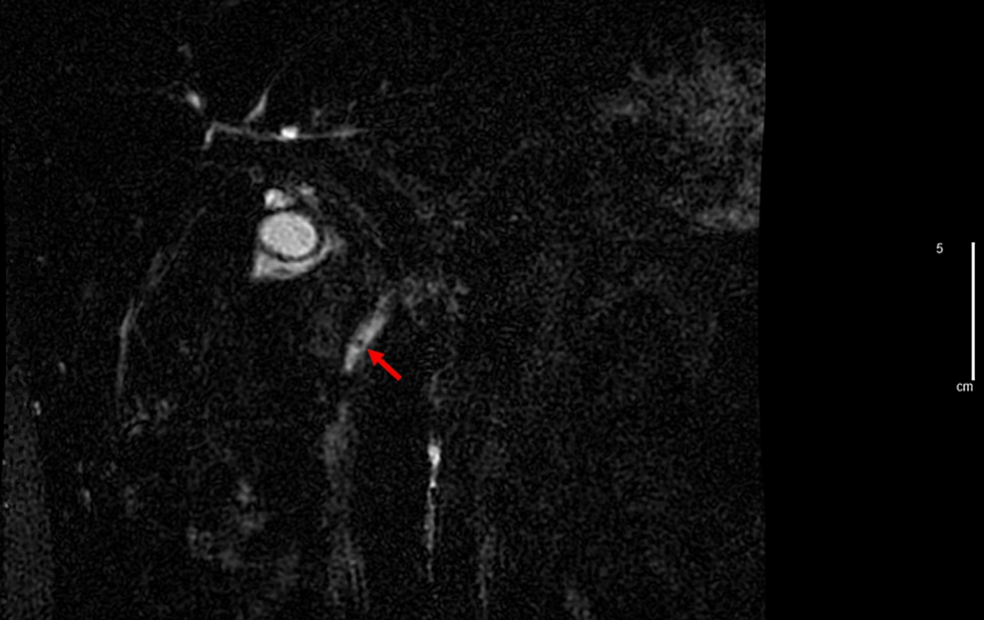
MRCP showing 0.4 cm stone in the common bile duct (red arrow) at 1.42x magnification. MRCP - magnetic resonance cholangiopancreatography

On presentation to the emergency department, his vitals were stable although he appeared uncomfortable from pain. His physical exam was significant for epigastric and RUQ abdominal tenderness on palpation but Murphy’s sign was negative, and he had no rebound tenderness or guarding. Initial labs were significant for hemoglobin of 11.1 g/dL (baseline is 11-12 g/dL) with sickled cells seen on the manual differential, LDH of 845 µ/L, total bilirubin of 8.1 mg/dL, direct bilirubin of 4.2 mg/dL, AST of 123 U/L, ALT of 68 U/L, and lipase was 15 µ/L. CT scan and RUQ ultrasound showed cholelithiasis, biliary sludge, gallbladder wall thickening, and a 0.6 cm dilated common biliary duct without evidence of stone or pancreatitis (Figure 1). The Gastroenterology team was consulted upon admission, and magnetic resonance cholangiopancreatography (MRCP) showed a 0.4 cm stone in the distal common bile duct (Figure 2).

**Figure 3:**
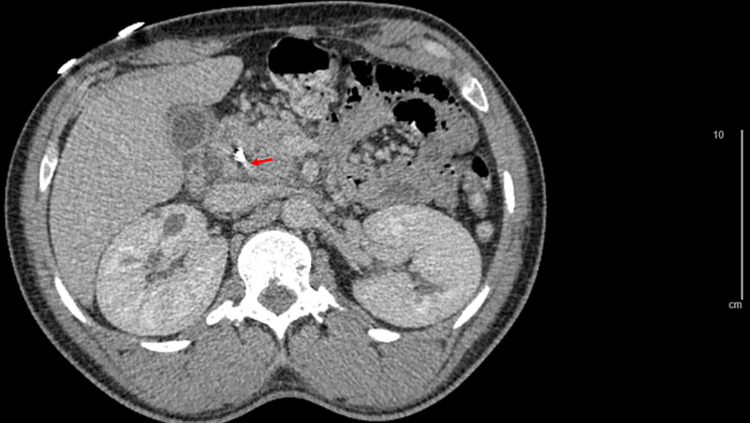
CT scan showing area of contrast blush concerning pseudoaneurysm (red arrow), and pancreatitis with peripancreatic free fluid collection at 1.42x magnification.

ERCP was subsequently performed with sphincterotomy, pancreatic duct cannulation, removal of black-pigmented stones, and placement of two stents in the bile ducts (8.5Fr straight plastic stents) and one in the pancreatic duct (5Fr single pigtail plastic stent). After the procedure, he developed significant mid-abdominal pain that felt different from his presenting symptoms. His labs were repeated and showed an acute drop in his hemoglobin from 11.1 g/dL to 5.9 g/dL. His lipase increased to 1,604 µ/L and amylase increased to 712 µ/L. CT angiogram showed acute pancreatitis without peripancreatic fluid collection and contrast blush near the duodenum in the proximity of the biliary stents (Figure 3). Mesenteric angiography by Interventional Radiology (IR) showed a pseudoaneurysm of a subsidiary vessel of the inferior pancreaticoduodenal artery (IPDA), which was injected with gel foam. The patient continued to have abdominal pain and a further decrease in his hemoglobin to 5.2 g/dL requiring coil embolization by IR with confirmation of no further active extravasation or pseudoaneurysm.

Over the next two days, the patient’s abdominal pain resolved, his lipase and amylase levels normalized, and his hemoglobin returned to baseline. He was discharged home with a follow-up for a cholecystectomy.

## Discussion

ERCP has been a critical tool to diagnose and treat patients with biliary pathology [7]. However, the risk for post-procedural pancreatitis can occur in up to 14% of patients [8]. Pancreatitis can then lead to the formation of pseudoaneurysms in 10% of cases which is a significant cause of morbidity and mortality from hemorrhage [4]. Pseudoaneurysms are missed 58% of the time so it should be considered when patients develop abrupt worsening of abdominal pain, acute decrease in hemoglobin, hemodynamic instability, and when there is clinical suspicion of gastrointestinal bleeding without obvious cause [4,9,10]. Several mechanisms have been proposed for this in pancreatitis, such as pancreatic enzyme digestion of the vessel wall, direct trauma to the vessel, mechanical irritation from post-operative drains, enlarging pseudocysts or abscesses causing ischemia with the release of pancreatic enzymes, and vessel wall stress [3,8,11]. These can result in localized bleeding, vessel erosion, and hemorrhage [3,11]. Our patient developed pancreatitis after ERCP from trauma secondary to instrumentation insertion, stent placement, or the contrast medium [8]. He subsequently developed acute worsening abdominal pain with a drop in his hemoglobin and was found to be bleeding into a pseudoaneurysm of the IPDA on angiography [12].

Our patient had many suspected mechanisms for pseudoaneurysm formation and we believe that his sickle cell disease may have contributed to his clinical picture. Although sickle cell disease can very uncommonly cause pancreatitis, our patient did not satisfy the Atlanta criteria for pancreatitis on admission [1,2,13]. He had a normal lipase level, no signs of pancreatitis on CT imaging, and abdominal pain that was different from his usual sickle cell crises pain. Intravascularly, sickled red blood cells can damage blood vessels in a vicious cycle described by vascular inflammation, stress, and ischemia from microvessel occlusion [5,13-15]. Sickled cells can also attach to the endothelial layer of vessels to cause fragmentation and degeneration of the smooth muscle layer as seen in sickle cell-related cerebral aneurysms [6]. Our patient, therefore, has vasculopathy based on his history, with prior splenic sequestration requiring splenectomy, several transient ischemic attacks but normal brain imaging, bilateral retinal artery occlusion, and monthly vaso-occlusive crises requiring hospitalizations.

The vasculopathy from our patient’s sickle cell disease likely caused an already compromised vessel wall at the IPDA, in addition to an active sickle cell crisis denoted by LDH two times his baseline, and sickled cells are seen on the manual differential, direct trauma during the ERCP, mechanical irritation from the stents, and post-procedure pancreatitis, all could have contributed to the formation of a pseudoaneurysm [15]. Similar phenomena of two patients with sickle cell disease and pseudoaneurysms have been reported in the literature: one within a tibial abscess and another within the IPDA as well [16,17]. This rare and recurrent association will need to be further explored as a potential risk factor for pseudoaneurysm formation in the sickle cell patient population.

Conventionally, pseudoaneurysms have been treated using surgical and endoscopic approaches [3,10,18]. An endovascular approach is now the first line for hemodynamically stable patients with as high as 100% success rate and 37% chance of recurrent bleeding [5,11]. This has led to a lower length of stay in the hospital and a decrease in the need for subsequent blood transfusions [5,11]. Options for management include gel foam slurries which can be injected; however, vessels can recanalize in several months so these may require further procedures or surgeries [19]. Alternatively, coils can be placed as well but distal migration can occur, so the decision is based on the operator [5]. The celiac artery and the proximal superior mesenteric artery branches such as the IPDA, in particular, are amenable to endovascular procedures because these vessels have adequate collaterals to maintain organ perfusion [5]. The main limitation with this approach is that the extent of the pseudoaneurysm cannot be completely assessed and bleeding might not always be detected [5]. This was likely why our patient needed coil embolization after gel foam slurry injection for hemostasis. Follow-up imaging is usually done 24 to 48 hours and one-month post-embolization [20].

## Conclusions

In conclusion, pseudoaneurysm rupture is an emergency and should be considered in patients who had prior biliary/pancreatic manipulation in the setting of active inflammation with an acute drop in hemoglobin, hemodynamic instability, and significant abdominal pain. There are multiple mechanisms in which pseudoaneurysms can form and we postulate that sickle cell disease is a possible contributor because of vascular wall stress and injury from vaso-occlusion. The management of pseudoaneurysms has shifted toward endovascular approaches because of better outcomes and minimally invasive techniques, but there is still a risk for residual bleeding requiring follow-up imaging.

## References

[1] Chatila AT, Bilal M, Guturu P (2019). Evaluation and management of acute pancreatitis. World J Clin Cases.

[2] Moori P, Dosis A, Ahmad Z, Kausar A, Triantafyllopoulou D (2018). Acute pancreatitis as a complication of sickle cell anaemia. Reports.

[3] White AF, Baum S, Buranasiri S (1976). Aneurysms secondary to pancreatitis. AJR Am J Roentgenol.

[4] Verde F, Fishman EK, Johnson PT (2015). Arterial pseudoaneurysms complicating pancreatitis: literature review. J Comput Assist Tomogr.

[5] Barge JU, Lopera JE (2012). Vascular complications of pancreatitis: role of interventional therapy. Korean J Radiol.

[6] Oyesiku NM, Barrow DL, Eckman JR, Tindall SC, Colohan AR (1991). Intracranial aneurysms in sickle-cell anemia: clinical features and pathogenesis. J Neurosurg.

[7] Pekgöz M (2019). Post-endoscopic retrograde cholangiopancreatography pancreatitis: a systematic review for prevention and treatment. World J Gastroenterol.

[8] Pezzilli R, Romboli E, Campana D, Corinaldesi R (2002). Mechanisms involved in the onset of post-ERCP pancreatitis. JOP.

[9] Vittoria De Martini I, Pfammatter T, Puippe G, Clavien PA, Alkadhi H (2020). Frequency and causes of delayed diagnosis of visceral artery pseudoaneurysms with CT: Lessons learned. Eur J Radiol Open.

[10] de Perrot M, Berney T, Bühler L, Delgadillo X, Mentha G, Morel P (1999). Management of bleeding pseudoaneurysms in patients with pancreatitis. Br J Surg.

[11] Sharma S, Prasad R, Gupta A, Dwivedi P, Mohindra S, Yadav RR (2020). Aneurysms of pancreaticoduodenal arcade: clinical profile and endovascular strategies. JGH Open.

[12] Rim D, Yu Q, Cieslak J, Wang W (2021). Bleeding pseudoaneurysm of the inferior pancreaticoduodenal artery as an endoscopic retrograde cholangiopancreatography complication. ACG Case Rep J.

[13] Ahmed S, Siddiqui AK, Siddiqui RK, Kimpo M, Russo L, Mattana J (2003). Acute pancreatitis during sickle cell vaso-occlusive painful crisis. Am J Hematol.

[14] Faes C, Sparkenbaugh EM, Pawlinski R (2018). Hypercoagulable state in sickle cell disease. Clin Hemorheol Microcirc.

[15] Saito N, Nadgir RN, Flower EN, Sakai O (2010). Clinical and radiologic manifestations of sickle cell disease in the head and neck. Radiographics.

[16] Cuthbert F, Gulati MS, Constantinescu G, Robertson P (2010). A pseudoaneurysm within a subperiosteal collection in a patient with sickle cell disease. J Clin Ultrasound.

[17] Gulati GS, Gulati MS, Makharia G, Hatimota P, Saikia N, Paul SB, Acharya S (2006). Percutaneous glue embolization of a visceral artery pseudoaneurysm in a case of sickle cell anemia. Cardiovasc Intervent Radiol.

[18] Nemakayala D, Ling X, Laird-Fick H (2017). Gastroduodenal artery pseudoaneurysm: a complication of pancreatitis. Am J Gastroenterol.

[19] Carr JA, Cho JS, Shepard AD, Nypaver TJ, Reddy DJ (2000). Visceral pseudoaneurysms due to pancreatic pseudocysts: rare but lethal complications of pancreatitis. J Vasc Surg.

[20] Madhusudhan KS, Venkatesh HA, Gamanagatti S, Garg P, Srivastava DN (2016). Interventional radiology in the management of visceral artery pseudoaneurysms: a review of techniques and embolic materials. Korean J Radiol.

